# Thermoresponsive Magnetic Hydrogel for Local Thermal Ablation Treatment of Rectal Cancer

**DOI:** 10.1021/acsami.5c22174

**Published:** 2026-01-02

**Authors:** Yuming Zhang, Christina Paraskeva, Isha Shaffir, Marco Tjakra, Vasiliki Koliaraki, Alexandra Teleki

**Affiliations:** † Department of Pharmacy, Science for Life Laboratory, 8097 Uppsala University, 75123 Uppsala, Sweden; ‡ Institute for Fundamental Biomedical Research, 54573 Biomedical Sciences Research Center ‘Alexander Fleming’, 16672 Vari, Greece; § Department of Pharmacy, Uppsala University, 75123 Uppsala, Sweden

**Keywords:** superparamagnetic iron oxide nanoparticle, magnetic hyperthermia, alternating magnetic field, critical gelation temperature, nanomedicine, doped ferrite, thermoresponsive polymer, flame spray pyrolysis

## Abstract

Thermal ablation treats cancer by raising local tumor temperature above ∼50 °C to induce coagulative necrosis. However, current approaches are invasive, as they typically require needle-like applicators to deliver thermal energy directly into the tumor. In this study, a magnetic hydrogel was developed as a noninvasive strategy for localized rectal cancer ablation aimed at preoperative tumor downsizing. The formulation comprises superparamagnetic iron oxide nanoparticles (SPIONs: Mn_0.6_Zn_0.4_Fe_2_O_4_) dispersed in Pluronic F127 (PF127). PF127 confers thermoresponsive sol–gel behavior at physiological temperature: upon contact with tissue, the formulation rapidly gels and becomes immobilized at the application site, minimizing spread to adjacent healthy tissue. After gel placement, an alternating magnetic field (AMF) is applied externally to activate the SPIONs to generate heat *in situ*, inducing local thermal ablation at the disease site. The gel can be administered either by syringe injection or by topical application, depending on tumor geometry. Adding SPIONs to the PF127 matrix did not alter the critical gelation temperature (CGT, 27.7 °C) but increased gel hardness and adhesiveness. When exposed to a 14 mT AMF at 596.2 kHz, the magnetic hydrogel exhibited excellent heating performance, reaching temperatures above the thermal ablation threshold (50 °C) within 3 min. The thermal ablation efficacy of the magnetic hydrogel was evaluated both *ex vivo* and *in vivo* using colorectal cancer xenograft mouse models. *Ex vivo* studies showed that a 15 min thermal ablation treatment resulted in an immediate 30% reduction in tumor volume. *In vivo*, a more pronounced effect was observed, with tumor volume decreased 69% 2 days after a single 20 min treatment. The finding highlighted the potential of the magnetic hydrogel as an alternative treatment for localized rectal cancer, either for preoperative tumor down-sizing or in cases where standard therapies such as surgery or chemo-radiotherapy are not feasible.

## Graphical abstract

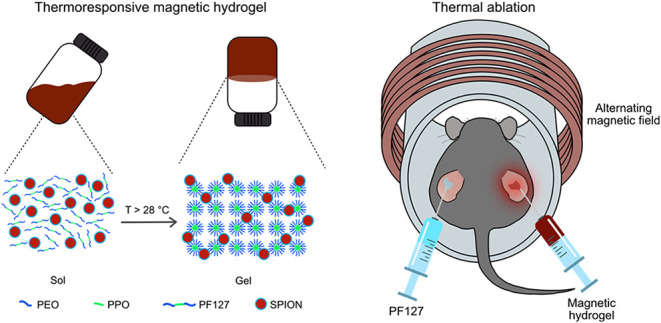

## Introduction

Colorectal cancer (CRC) is the second most frequently diagnosed cancer and the second leading cause of cancer-related death in the European Union.^1^ Rectal cancer accounts for approximately 35% of all CRC cases.^2^ Standard management is stage-dependent and typically includes local excision with adjuvant or neoadjuvant chemoradiotherapy to downsize tumors and improve resectability, particularly in stage II–III disease.^3^ However, several weeks of chemoradiation can substantially diminish patients’ quality of life prior to surgery, and a considerable proportion of patients develop resistance during treatment. In addition, some tumors are considered surgically inaccessible because of proximity to critical pelvic structures or patient comorbidities.

Thermal ablation is an alternative local treatment that eradicates malignant tissue by elevating intratumoral temperature (typically ≥ 50 °C). Common clinical modalities include radiofrequency, microwave, laser, and focused ultrasound ablation, delivered via image-guided, needle-like applicators positioned within the tumor.^4^ While effective and applicable in certain tumors (e.g., colorectal liver metastases),^5^ these probe-based approaches are not used for treating localized rectal tumors in the clinic due to procedural invasiveness, the need for precise device placement and patient immobilization, and risks of off-target injury from excessive heating (e.g., transmural bowel injury).^6^ These challenges motivate the development of less invasive alternatives that generate heat locally without insertion of rigid applicators.

Magnetic thermal ablation is a minimally invasive or noninvasive therapeutic approach in which superparamagnetic iron oxide nanoparticles (SPIONs) are activated by an external alternating magnetic field (AMF) to generate *in situ* heat within the nanoparticle-laden region. The heat produced under AMF can raise local tumor temperature to the ablation threshold (>50 °C), inducing cancer cell death primarily via coagulative necrosis.^4,7^ Heating efficiency can be enhanced by compositional tuning of iron-oxide cores: manganese and zinc doping (alone or combined) often increases saturation magnetization and improves heating performance compared with undoped particles.^8–11^ From a safety standpoint, the established clinical use of SPIONs as MRI contrast agents and their clinical application in cancer hyperthermia support a favorable biocompatibility profile and translational potential.^12–16^

However, administering SPIONs as aqueous suspensions is suboptimal. Delivery route is limited to intratumoral injection, which is invasive and difficult to control in terms of distribution and retention. Intratumoral injection has been reported to yield inhomogeneous distribution,^17,18^ and liquid suspensions are prone to backflow/leakage along the needle tract, restricting local retention and increasing the risk of broad systemic distribution and systemic exposure. Thus, embedding SPIONs within a retentive, tissue-adherent matrix is preferred.

Thermoresponsive hydrogels offer an attractive formulation matrix due to their injectability, biocompatibility, and rapid gelation at body temperature. Among these, Pluronic F127 (PF127) is widely used and is an FDA-approved excipient in pharmaceutical products, including ophthalmic products such as AzaSite and Besivance. Its thermoresponsive behavior enables a reversible sol–gel transition at a concentration-dependent critical gelation temperature (CGT).^19,20^ By formulating SPIONs in PF127 with a physiologically relevant CGT (≤37 °C), the mixture can be administered as a low-viscosity liquid via injection or topical application and then gels *in situ* on contact with tissue, immobilizing SPIONs locally. However, the inclusion of additives like SPIONs may influence PF127 self-assembly and shift its sol–gel transition temperature,^21^ making it essential to characterize the gelation properties of the final hydrogel formulation to ensure that it maintains a CGT within the physiological range. Overall, this hydrogel system promotes uniform tumor coverage, enhances intratumoral retention, and reduces leakage into adjacent healthy tissue or the rectal lumen.^22^

This study developed a PF127 hydrogel formulation loaded with SPIONs, referred to as magnetic hydrogel. The formulation is liquid at room temperature and rapidly gels at physiological temperature upon contact with tissue, enabling localized thermal ablation of rectal tumors. It permits minimally invasive delivery via transanal intratumoral injection, with *in situ* gelation that immobilizes the SPIONs and reduces leakage, or by topical application to conformally coat irregular tumor surfaces. Although hydrogels combining PF127 and SPIONs have been proposed for cancer hyperthermia, *in vivo* evaluation has not yet been reported.^23,24^ Accordingly, the present study evaluates the magnetic hydrogel’s thermoresponsive behavior, delivery by injection and topical routes, mechanical and bioadhesive properties, heating performance under an AMF, and ablation outcomes in CRC models *ex vivo* and *in vivo*.

## Results and Discussion

### Flame Synthesis of SPIONs and Toxicity

Silica-coated manganese- and zinc-doped superparamagnetic iron oxide nanoparticles (SPIONs; SiO_2_-coated Mn_0.6_Zn_0.4_Fe_2_O_4_) were selected for their superior heating efficiency under AMF compared with undoped iron oxides.^8–11^ These SPIONs were synthesized by flame spray pyrolysis, a scalable process that enables precise control of size, composition, and silica coating in line with pharmaceutical quality-by-design manufacturing.^9,25^ The synthesis and detailed physicochemical characterization of these flame-made SPIONs have been reported previously.^8^ Briefly, the particles had an average crystallite size of about 15 nm and a surface silica coating that rendered them hydrophilic (specific surface area 52 m^2^ g^–1^ in agreement with literature,^26^ ζ-potential ≈ −40 mV; Table S1), facilitating dispersion in the hydrogel. Their saturation magnetization and specific absorption rate were 94.8 emu g^–1^ and 125 W g^–1^, respectively, indicating high magnetization and efficient heating during AMF exposure, which supports their use in AMF-induced thermal ablation.^8^

Establishing the safety of formulation components is a prerequisite for clinical translation. SPION safety was therefore evaluated using both *in vitro* and *in vivo* models. PF127, the polymer used in the magnetic hydrogel, is FDA-approved and widely used in pharmaceutical products, with a well-established safety profile. Therefore, its toxicity was not assessed in this study.

The cytotoxicity of SPIONs was assessed in three human CRC cell lines, SW480, HT29, and Caco-2, all commonly used in CRC preclinical research (Figure 1A). Cell viability decreased with increasing particle concentration, especially for HT29 and Caco-2 at >0.6 mg/mL SPION. However, all cell lines maintained ≥70% viability at the highest SPION concentration tested (1.0 mg mL^–1^), meeting the ISO 10993–5 criterion for noncytotoxicity. Biocompatibility is attributed to the presence of a silica surface layer that acts as a barrier around the iron-oxide core, thereby reducing toxicity.^27^ *In vivo* safety was further assessed using a zebrafish embryo model. Embryos in all treatment groups exhibited morphology comparable to PBS controls (Figure 1B), as confirmed by quantitative analysis of anatomical regions (Figures S1 and 1C). No significant toxicity was observed up to 5 days postfertilization (pdf), as indicated by normal body shape, eye size, pericardial area, and successful inflation of the swim bladder. These findings demonstrate that SPIONs did not induce developmental toxicity in the zebrafish model.

**Figure 1:**
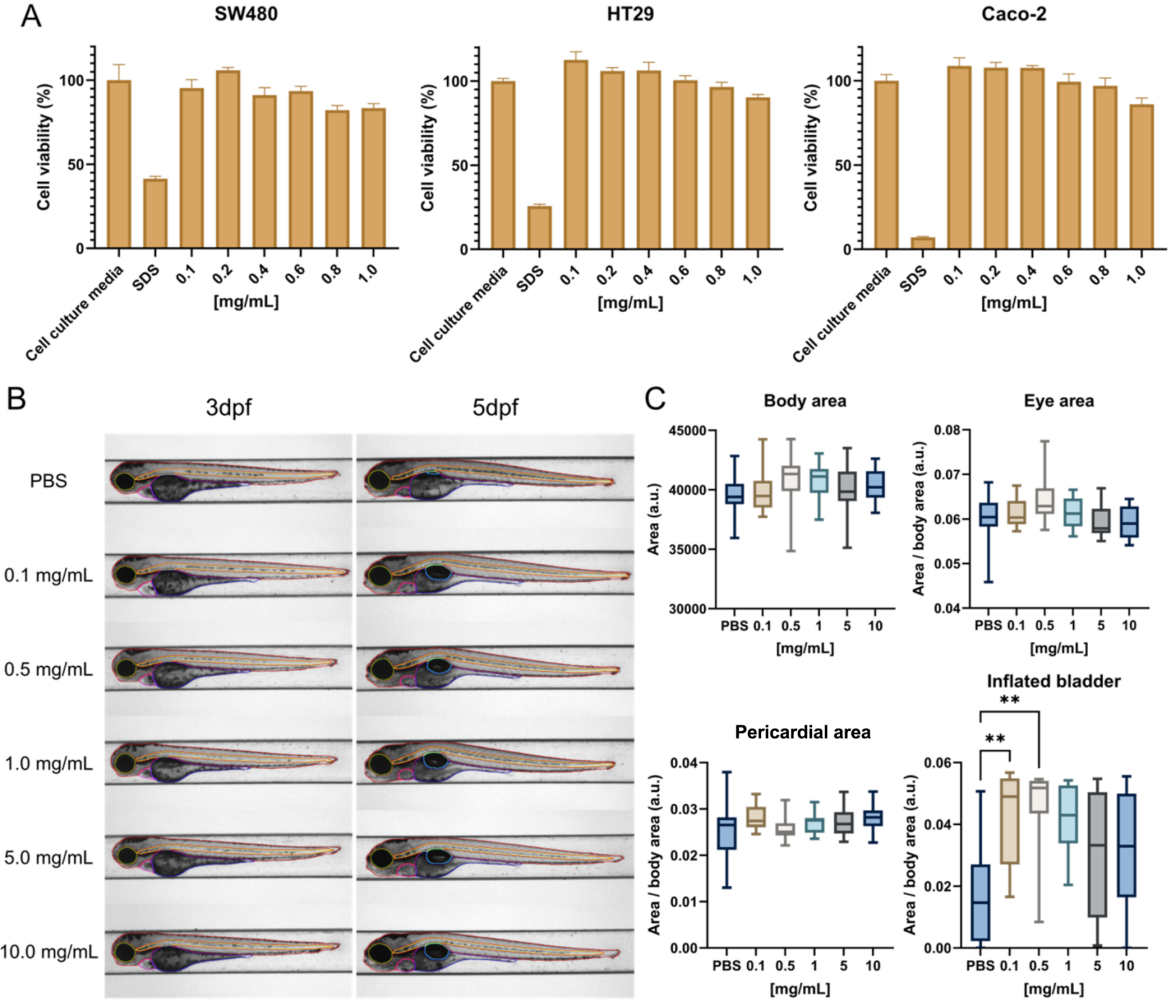
Toxicity profile of SPIONs using *in vitro* cell models and *in vivo* zebrafish embryos. (A) Cell viability of SPIONs in CRC cell lines SW480, HT29 and Caco-2 in a concentration-dependent manner (from 0.1 to 1.0 mg/mL). Cell culture media and 0.22% (v/v) sodium dodecyl sulfate (SDS) were used as negative and positive controls, respectively (*n* = 5). (B) Representative lateral-view images of zebrafish embryos that were injected with SPION suspensions (0.1–10.0 mg/mL) at 2 days dpf, and imaged at 3 and 5 days pdf. PBS-injected embryos served as controls. Anatomical regions were highlighted as follows: body area (red), eye area (green), pericardial area (pink), and swim bladder (blue). (C) Quantification of body, eye, and pericardial areas at 3 dpi (**p ≤ 0.01; *n* ≥ 12). All data are expressed as mean ± standard deviations.

Overall, SPIONs exhibited no detectable toxicity in either CRC cell lines or the zebrafish embryos, underscoring their acceptable safety profile and supporting their translational potential for clinical application.

### Magnetic Hydrogel Characterization

The hydrogel matrix is formed from the FDA-approved thermoresponsive polymer PF127, which consists of polar poly­(ethylene oxide) (PEO) blocks and nonpolar poly­(propylene oxide) (PPO) blocks arranged in a triblock structure. At the CGT, these triblocks self-assemble into an organized micellar network, resulting in a sol-to-gel transition (Figure 2A), which underlies the thermoresponsive property of PF127. SPIONs are distributed within the PF127 matrix to form the magnetic hydrogel. For *in vivo* application, the magnetic hydrogel should remain in the sol state at room temperature to facilitate administration and undergo gelation at physiological temperature once in contact with the tumor tissue (Figure 2B). Consequently, the CGT must fall between room temperature and physiological temperature to ensure both ease of administration and retention at the target site.

**Figure 2:**
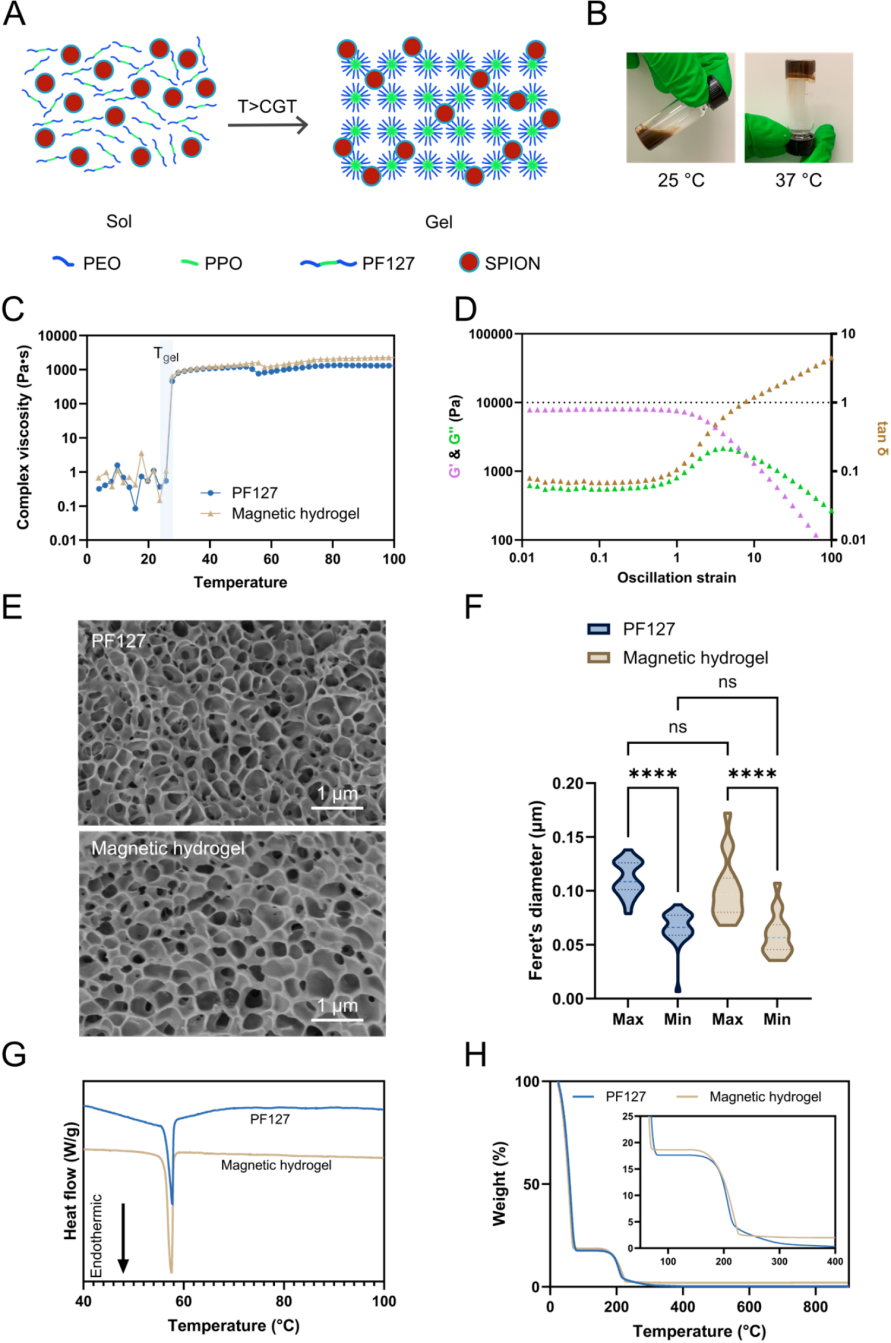
Thermoresponsive and rheological profile of the magnetic hydrogel. (A) Schematic illustration of the sol–gel transition process of magnetic hydrogel. (B) Representative images of magnetic hydrogel at sol state at 25 °C (left) and gel state at 37 °C (right). (C) Complex viscosity as a function of temperature at a constant oscillation strain of 0.1% for the PF127 (blue symbols) and magnetic hydrogels (brown symbols). (D) Oscillatory amplitude sweep of magnetic hydrogel at 37 °C at a constant frequency of 1 Hz. (E) Cryo-SEM images of the PF127 hydrogels (upper panel) and magnetic hydrogel (lower panel). (F) Feret’s maximum and minimum diameter of the two hydrogel formulations. The dashed line in the middle of the violin plot represents the median, and the dashed lines on the sides represent the quartiles (*n* ≥ 16). (G) DSC thermograms and (H) TGA profiles of PF127 (blue line) and magnetic hydrogel (brown line). (ns*p >* 0.05, ****p ≤ 0.001).

The CGT of pure PF127 and the magnetic hydrogel was determined by rheological temperature sweeps. Figure 2C shows the complex viscosity of both formulations as a function of temperature. In both cases, the sol–gel transition was marked by a sharp increase in viscosity, initiating at 25.7 °C and completing at 27.7 °C (*n* = 3). This indicates that both formulations remain in the sol state below 25.7 °C and are fully gelled by 27.7 °C. The identical CGT values demonstrate that SPION incorporation did not affect the gelation process of PF127, suggesting that the nanoparticles are accommodated within the interstitial spaces of the micellar lattice without disrupting the PEO–PPO–PEO self-assembly or micellar packing that governs gelation.

The complex viscosity for both formulations stayed above 1000 Pa·s from 50 °C to 100 °C, which corresponds to the thermal ablation temperature range (*G*′ and *G*″ for the temperature sweep are shown in Figure S2). This demonstrates that the formulations maintain their gel structure during thermal ablation, thereby ensuring that the magnetic hydrogel remains localized within the tumor tissue during treatment.

The viscoelastic behavior of the magnetic hydrogel (Figure 2D) and PF127 (Figure S3) at 37 °C were further analyzed under varying shear stress. Both *G*′ and *G*″ were comparable between the magnetic hydrogel and PF127 hydrogel, indicating that SPION incorporation did not weaken the viscoelastic network of PF127. At low oscillation strains (0.01–1%), both formulations exhibited a plateau in tan δ, defining the linear viscoelastic region (LVR). Beyond this plateau, tan δ gradually increased, reflecting a shift from elastic (gel-like) to viscous (sol-like) dominance. The transition was completed at an oscillation strain of 6.2% for PF127 and 7.9% for the magnetic hydrogel. The slightly lower transition strain of the PF127 suggests it is more brittle compared to the magnetic hydrogel.

The morphology of the hydrogels was visualized by cryo-scanning electron microscopy (SEM), revealing similar porous structures in both formulations (Figure 2E). Feret’s diameter analysis showed no significant differences between the magnetic hydrogel and pure PF127 hydrogels, indicating that SPION addition did not alter the microstructure of PF127 (Figure 2F). Moreover, the differences observed between the maximum and minimum Feret’s diameters for both formulations suggest noncircular pore geometry, which is visually apparent in the SEM images and consistent with literature.^28^

The thermograms of PF127 and magnetic hydrogel were analyzed by differential scanning calorimetry (DSC) to assess whether SPION incorporation altered the crystallinity and thermal behavior of PF127. Both formulations exhibited a sharp endothermic peak at ∼57 °C (*n* ≥ 3), indicating that the crystallinity of PF127 and its characteristic thermal transitions remain unchanged in the presence of SPIONs (Figure 2G).^29^ This peak corresponds to two overlapping processes: (i) melting of semicrystalline PEO domains and (ii) endothermic dehydration of PEO blocks, both of which occur in this temperature window.^30–32^ The same thermal events were also observed in rheological measurements, where a slight decrease in complex viscosity was obtained at 55.8 °C for the magnetic hydrogel and 57.8 °C for PF127 (Figure 2C). The viscosity drop reflects a microscopic transition from a hard gel to a soft gel. At temperatures above ∼57 °C, melting of PEO crystallites decreases the crystalline reinforcement of the network, while simultaneous dehydration of PEO blocks reduces micellar corona overlap and entanglement density, together leading to gel softening.^31,32^

Figure 2H shows the thermogravimetric degradation profiles of PF127 and the magnetic hydrogel, which showed no notable differences. In both formulations, an initial sharp weight loss from room temperature to approximately 77 °C was attributed to water evaporation. A second major weight loss between approximately 166 °C and 226 °C corresponded to the thermal degradation of the PF127 polymer. The residual mass weight of 2.0 wt % at 900 °C in the magnetic hydrogel was attributed to the inorganic SPION content, consistent with the theoretical value of 2 wt %.

### Injectability, Tissue Adhesion, and Texture Analysis of Magnetic Hydrogel

For clinical use, the magnetic hydrogel can be administered either by injection through a needle or applied topically to the tumor surface, the latter being particularly useful for superficial or irregularly shaped tumors where injection may be challenging. As shown in Figure 3A, the magnetic hydrogel is injectable in both sol and gel states. In the sol state, it behaves as a free-flowing liquid that can be easily extruded through a 26-gauge needle, while in the gel state it can still be smoothly extruded through the same needle. This is clinically beneficial because even if gelation occurs prior to injection, for example, due to prolonged handling by the clinician, the material remains injectable. For topical application, Figure 3B shows the gel applied to the luminal side of porcine rectal tissue, where it remained firmly attached even when the tissue was held vertically, demonstrating strong tissue adhesion.

**Figure 3:**
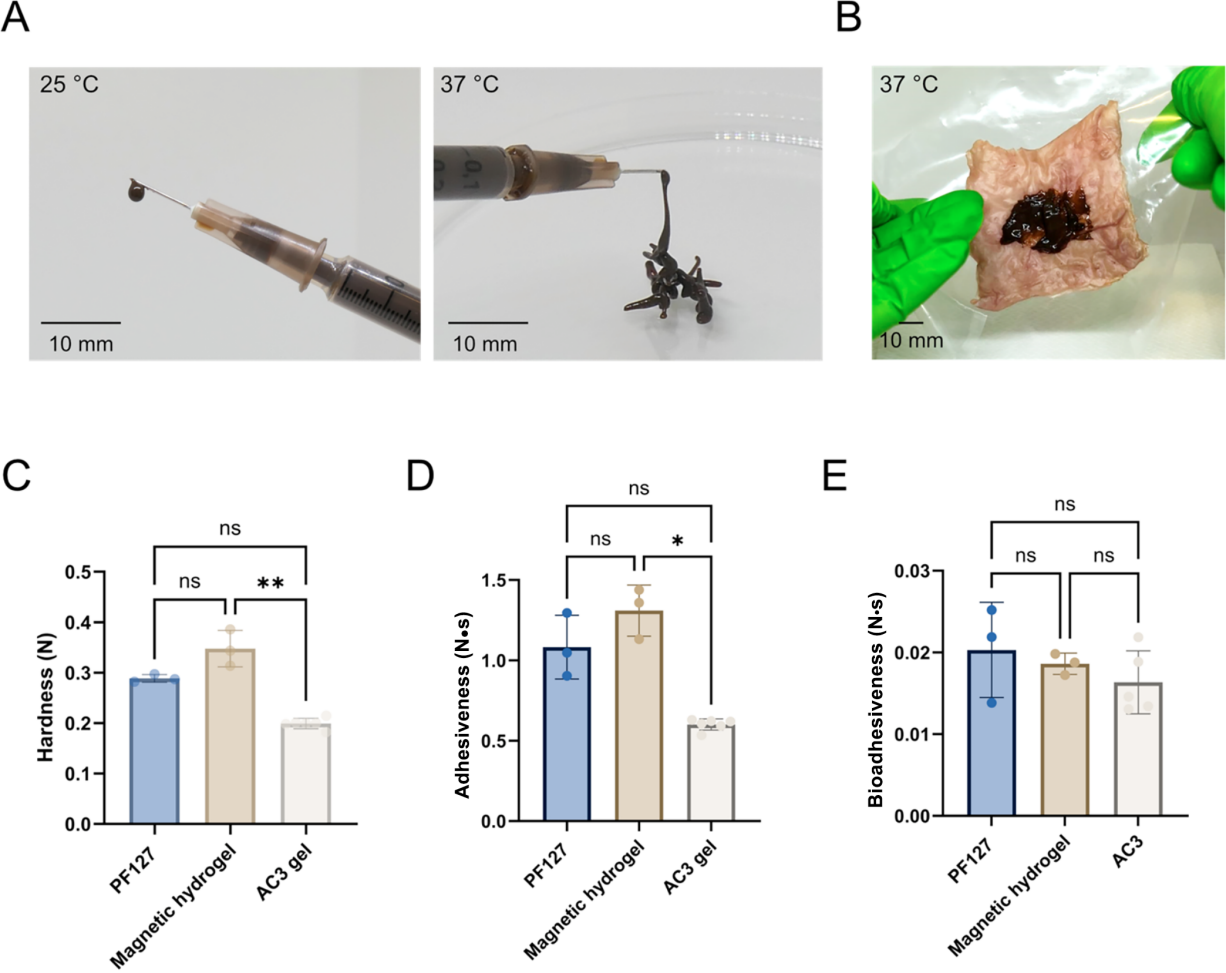
Mechanical properties and bioadhesiveness of PF127 gel, magnetic hydrogel and commercial rectal topical AC3 gel. (A) Representative images showing magnetic hydrogel extruded through a 26-gauge needle in sol state at 25 °C (left) and in gel state at 37 °C (right). (B) Magnetic hydrogel gelled on a piece of porcine rectal tissue at 37 °C. (C) Hardness and (D) adhesiveness of hydrogel formulations. (E) Bioadhesiveness of hydrogel formulations when applied on porcine rectal tissues. All data are expressed as mean ± standard deviations (*n* ≥ 3). (ns*p >* 0.05, *p ≤ 0.05).

To further evaluate its mechanical properties, the gels were analyzed using a texture profile analyzer with a commercial rectal gel (AC3, used for topical hemorrhoid treatment) serving as a benchmark. As shown in Figure 3C,D, the magnetic hydrogel formulations exhibited greater hardness and adhesiveness than AC3, indicating their suitability as topically applied gels for local rectal tumor treatment. Incorporation of SPIONs into PF127 significantly enhanced both properties compared to the AC3 control. Similar improvements in mechanical properties upon SPION incorporation have been reported previously, typically attributed to SPION acting as rigid fillers that reinforce the polymeric network.^33^ For topical application, greater hardness increases hydrogel robustness and resistance to deformation under peristaltic force, while enhanced adhesiveness ensures firm and prolonged contact with tumor surfaces.

To mimic *in vivo* topical application, a bioadhesion test was conducted using porcine rectal tissue (Figure 3E). Bioadhesiveness was quantified as the work of adhesion during the detachment of tissue from the gel formulations. No significant differences were observed among the three formulations, indicating that the magnetic hydrogels have tissue adhesion comparable to the commercial AC3 gel, further supporting their suitability for topical use.

### *In Vitro* Heating Performance of Magnetic Hydrogel

The heating performance of the magnetic hydrogel was evaluated under a 30 min exposure to an external AMF. The effect of SPION concentration (5, 10, 15, and 20 mg/mL) on heating efficiency was first investigated (Figure 4A). Heating performance progressively increased with SPION concentration as expected. After 30 min of AMF exposure, the temperature increase (Δ*T*) for hydrogels containing 5, 10, 15, and 20 mg/mL SPIONs reached final temperatures of 58.6 °C, 76.6 °C, 85.4 °C, and 99.0 °C, respectively. All formulations exceeded the thermal ablation threshold (≥50 °C) within 10 min of AMF exposure. Based on these results, the 20 mg/mL SPION hydrogel was selected for further experiments due to its superior heating efficiency. Figure 4B shows the volume-dependent heating performance of the magnetic hydrogel containing 20 mg/mL SPIONs. Heating efficiency increased with hydrogel volume, and even the smallest tested volume (100 μL) reached thermal ablation threshold within 3 min. The small test volumes were chosen to align with downstream *in vivo* studies, given the limited intratumoral dosing capacity of mouse xenograft tumors. Overall, the magnetic hydrogel demonstrated excellent heating performance, highlighting its strong potential as a thermal ablation agent in cancer therapy.

**Figure 4:**
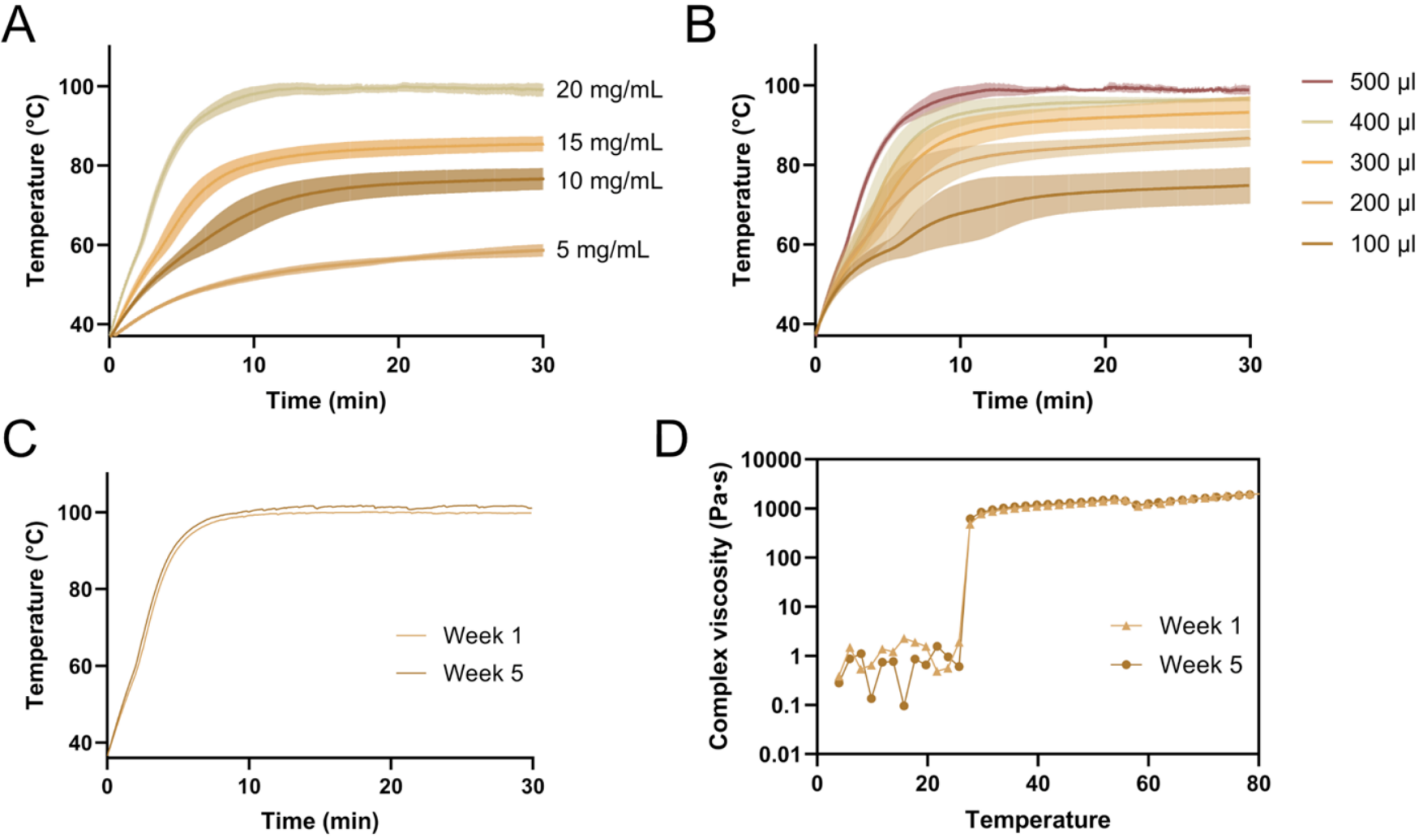
Heating performance and storage stability of the magnetic hydrogel. (A) Heating curves of 500 μL magnetic hydrogel samples containing different SPION concentrations during 30 min of AMF exposure. (B) Heating curves of magnetic hydrogel with 20 mg/mL SPIONs at varying sample volumes. Short-term stability of the magnetic hydrogel assessed by (C) heating performance and (D) rheological profile over 5 weeks of storage at 4 °C. AMF parameters: *f* = 592.3 kHz, H = 14 mT. All data are expressed as mean ± standard deviations (*n* = 5 for A, *n* = 3 for B and *n* = 5 for C).

The short-term stability of the magnetic hydrogel was evaluated by monitoring its heating performance and rheological properties after 5 weeks of storage at 4 °C. Heating performance remained consistent after storage (Figure 4C), and no changes in gel viscosity were detected (Figure 4D). These findings demonstrate that the magnetic hydrogel exhibits good physicochemical stability under refrigerated conditions. Future studies should also characterize stability under higher storage temperature, such as room temperature, and longer storage periods to fully define its shelf life and handling requirements for clinical use.

### *Ex Vivo* and *In Vivo* Thermal Ablation of Tumors

The therapeutic effect of magnetic hydrogel-induced thermal ablation was first evaluated *ex vivo*. Tumors freshly excised from CRC xenograft mouse models were applied with either magnetic hydrogel or PF127 hydrogel (control), and then exposed to an AMF for 15 min, reaching saturation temperatures of approximately 80 °C. This temperature exceeds the threshold at which soft cancer tissue reportedly loses most of its water content (70–75 °C).^32^ To preserve tissue viability, treatments were conducted at the air–liquid interface using a transwell setup (Figure 5A).

**Figure 5:**
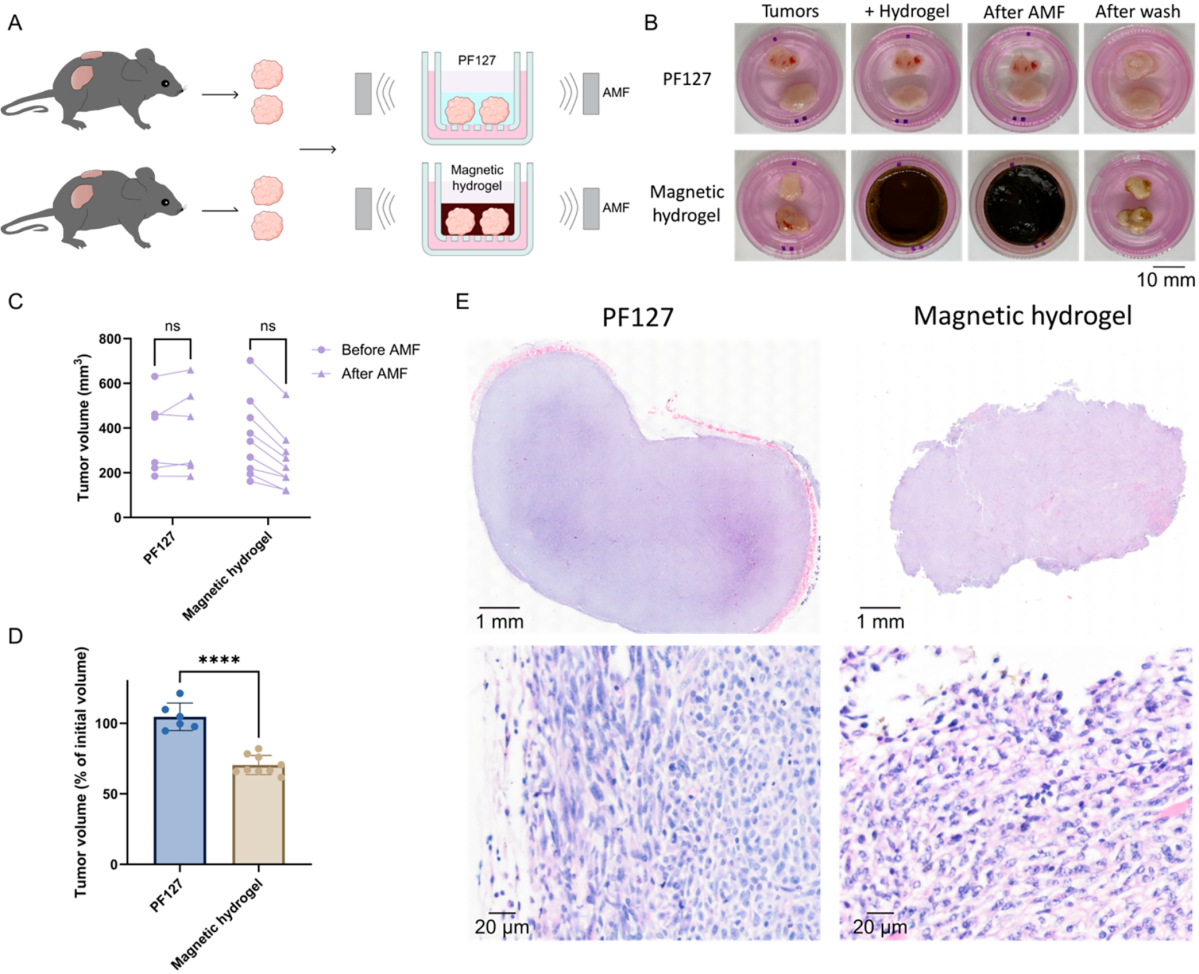
*Ex vivo* tumor thermal ablation using magnetic hydrogel. (A) Schematic illustration of the thermal ablation treatment on mouse tumors. Tumors were excised from mice, pooled by their size similarity, and randomly placed in transwell inserts to establish an air–liquid interface. Each tumor was then treated with either PF127 hydrogel or magnetic hydrogel, followed by 15 min of AMF exposure. (B) Representative images of tumors at key experimental stages: before treatment, after hydrogel application, post-AMF exposure, and after PBS wash. (C) Tumor volume before and after treatment with PF127 or magnetic hydrogel. (D) Tumor volume after treatment, normalized to initial volume. (E) H&E staining of the tumor treated with PF127 or magnetic hydrogel. (*n* ≥ 6, data in D were expressed as mean ± standard deviation). (ns*p >* 0.05, ****p ≤ 0.001).

Tumors exposed to the magnetic hydrogel under AMF exhibited a noticeably firmer and stiffer texture after treatment, indicative of protein denaturation, dehydration, and coagulative necrosis,^34,35^ whereas PF127-treated tumors showed no detectable change (Figure 5B). All magnetic hydrogel-treated tumors also showed an immediate reduction in size, while PF127-treated tumors remained unchanged (Figure 5C). Quantitative analysis confirmed a significant 30% decrease in tumor volume following magnetic hydrogel-induced thermal ablation compared to the PF127 control (Figure 5D), demonstrating a pronounced therapeutic effect even after a single 15 min AMF exposure.

Histological analysis using hematoxylin and eosin (H&E) staining revealed pronounced structural alterations in tumors subjected to thermal ablation (Figure 5E). Treated tumors displayed irregular morphology, poorly defined borders, and loss of the outer margin, consistent with surface cell damage or loss, likely caused by direct contact with the hot magnetic hydrogel. Internally, tumors were less dense and more heterogeneous, with increased intercellular spacing and altered cellular morphology, features indicative of adhesion loss and potential cell death. These histological changes likely reflect a combination of thermal ablation–induced cytotoxicity and dehydration.

The therapeutic efficacy of magnetic hydrogel-induced thermal ablation was further evaluated *in vivo* using the same CRC xenograft mouse model as in the *ex vivo* experiments. Injection was selected as the administration route to ensure reliable gelation within the tumor. In xenograft models, the tumor surface is exposed to the environment, creating temperature gradients that hinder uniform gelation of a topically applied hydrogel,^36^ unlike the stable 37 °C conditions of the rectum or colon. In addition, because xenograft tumors are subcutaneous, topical application risks damaging the overlying skin and causing animal discomfort. Thus, intratumoral injection was considered the most appropriate method for evaluating therapeutic efficacy *in vivo*.

Seven to 9 days after tumor inoculation, when tumors reached ∼7–10 mm in length, PF127 or magnetic hydrogel was injected into either of the two tumors on the back of each mouse. Immediate gelation of the hydrogels occurred upon injection. The mice were positioned at the center of the coil and an AMF was applied for 20 min. After the treatment, the animals were housed for an additional 2 days before sacrifice, and tumor volumes were measured at the end point (Figure 6A).

**Figure 6:**
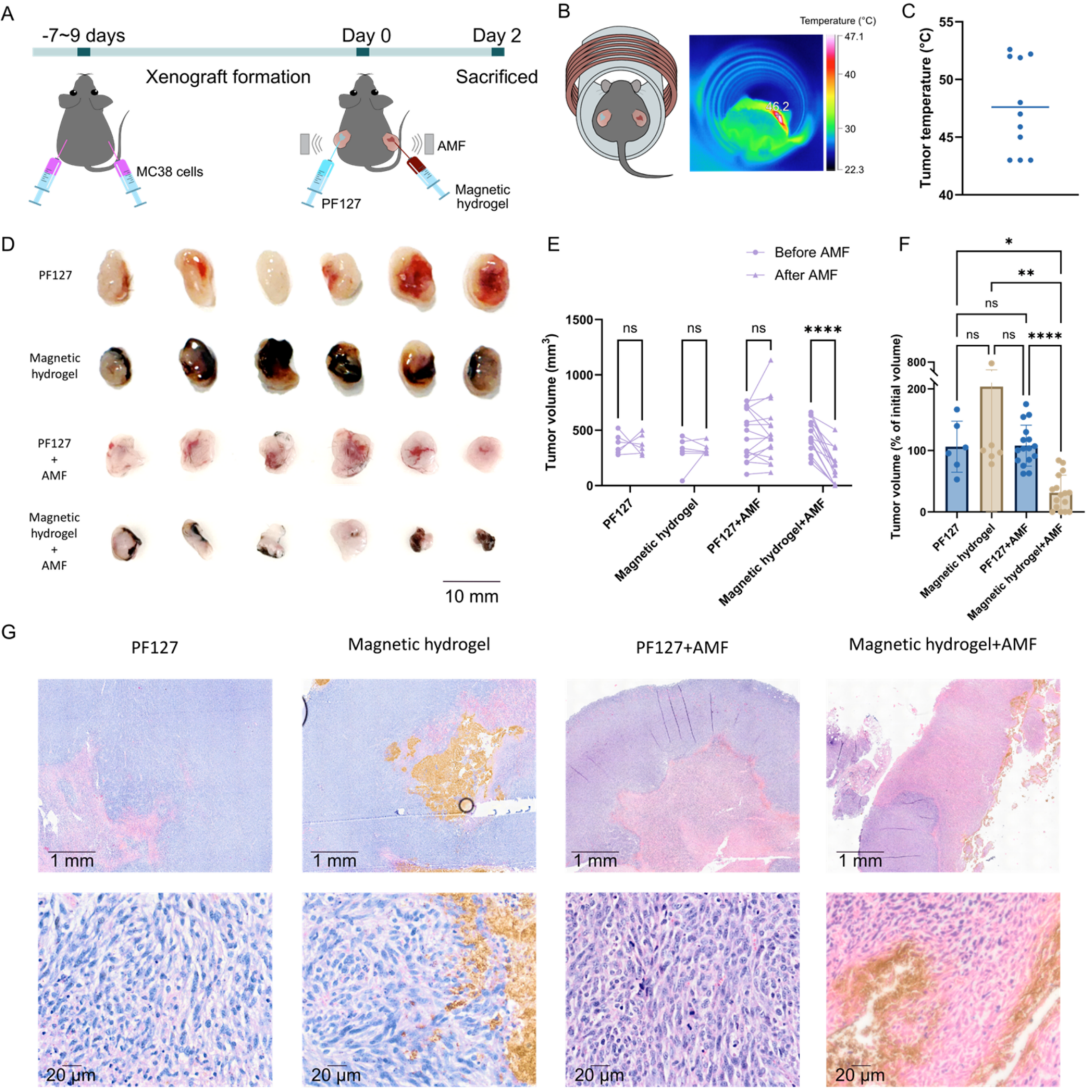
*In vivo* tumor thermal ablation using magnetic hydrogel. (A) Schematic of the thermal ablation treatment protocol in CRC xenograft mouse models. MC38 cells were injected into both flanks 7–9 days before treatment. On Day 0, tumors received either PF127 (control) or magnetic hydrogel, followed by 20 min of AMF exposure. Tumor volumes were measured on Day 0 and Day 2 (prior to sacrifice). (B) Experimental setup showing a mouse positioned inside the AMF coil (left) and a thermal image during AMF exposure (right), revealing localized heating only in magnetic hydrogel-treated tumors. (C) Average surface temperature of magnetic hydrogel-injected tumors under AMF exposure (*n* = 11). (D) Representative tumor images from each treatment group: PF127, PF127 + AMF, magnetic hydrogel, and magnetic hydrogel + AMF. (E) Tumor volume for all groups before (Day 0) and after AMF exposure (Day 2). (F) Tumor volumes on Day 2 expressed as percentage of initial volume (*n* ≥ 6 for E and F; AMF groups: females = 7, males = 9; control groups without AMF: female = 1, males = 5). (G) Representative H&E-stained tumor sections showing necrotic changes in the PF127, PF127 + AMF, magnetic hydrogel and magnetic hydrogel + AMF groups. Data in C, E, and F are presented as mean ± standard deviation. (ns*p >* 0.05, *p ≤ 0.05, **p ≤ 0.01, ****p ≤ 0.001).

Tumor temperatures during AMF exposure were monitored via infrared thermal imaging. The recorded temperatures represent only the tumor surface temperature, and internal tumor temperatures are likely higher. Tumors injected with magnetic hydrogel exhibited localized heating under AMF, while PF127-injected tumors showed no measurable temperature increase compared with surrounding tissue (Figure 6B). The average surface temperature of magnetic hydrogel-injected tumors reached 47.6 °C under AMF (Figure 6C).

Representative images of tumors 2 days post-treatment are shown in Figure 6D. Tumors treated with magnetic hydrogel and AMF (thermal ablation group) were visibly smaller than those in the control groups (PF127 alone, magnetic hydrogel alone, and PF127 + AMF). Quantitative analysis confirmed a significant decrease in tumor volume in the thermal ablation group, while no significant changes were observed in the controls (Figure 6E). When tumor volumes were normalized to their initial sizes, only the thermal ablation group showed a significant reduction (Figure 6F), with a 69% decrease in tumor volume. No differences in tumor volume were observed among the three control groups, indicating that neither PF127, magnetic hydrogel alone, nor PF127 under AMF exposure adversely affected tumor growth. Notably, other studies have reported that thermal ablation using similar SPION-based gels can lead to pronounced bleeding and inflammation, phenomenon that were not observed in our case.^37^ This variation likely reflects differences in experimental conditions such as mouse model, injection approach, or treatment parameters.

Histological analysis was performed to assess tissue damage induced by thermal ablation (Figure 6G). Tumors injected with magnetic hydrogel and exposed to AMF displayed distinct necrotic features compared with controls, including predominant eosinophilic (pink) staining corresponding to cytoplasmic and extracellular matrix components. Hematoxylin (blue) staining was markedly reduced, indicating loss of nuclei (karyolysis), along with small, condensed nuclei characteristic of early necrosis (pyknosis). Similar histological features were reported by Zhang and Song following 25 min of magnetic thermal ablation in mouse xenografts.^37^ Control tumors (PF127, magnetic hydrogel alone, and PF127 + AMF) also exhibited areas of central necrosis; however, this is a common feature of large xenograft tumors and thus unrelated to the treatment.^38^

The organ biodistribution in Figure S4 showed a higher iron accumulation in the spleen of thermally ablated mice compared to nontreated controls, while no significant differences were observed in the liver or kidneys. This finding is consistent with previous reports identifying the spleen as a major organ involved in iron oxide nanoparticle elimination.^39^

Overall, this *in vivo* mouse xenograft study demonstrated that a single 20 min magnetic hydrogel-induced thermal ablation session effectively reduced tumor size and induced tumor necrosis. Future experiments should assess additional and long-term effects of tumor thermal ablation, including the development of inflammation and potential tumor recurrence. Inflammatory responses are particularly relevant, as ablation-induced tissue damage can elicit a substantial local immune reaction that may either enhance antitumor immunity or promote tumor growth through mitogenic inflammatory mediators.^40^ Future investigations should also assess both injectable and topically applied magnetic hydrogels in orthotopic tumor models, and ultimately in large animal models with gastrointestinal anatomy more comparable to humans, such as pigs, to establish the translational feasibility of this therapeutic strategy.

## Conclusion

A magnetic hydrogel composed of 20 mg/mL SiO_2_-coated Mn_0.6_Zn_0.4_Fe_2_O_4_ and 22 wt % PF127 was successfully manufactured. PF127 exhibited thermoresponsive behavior, undergoing a sol–gel transition at 27.7 °C. The magnetic hydrogel was liquid at room temperature and gelled upon contact with tissue at physiological temperature. Incorporation of SPIONs did not alter the CGT of PF127 but enhanced its mechanical properties, including hardness and adhesiveness, making it suitable for topical application. The magnetic hydrogel also exhibited bioadhesiveness comparable to a commercial rectal gel. Furthermore, the gel could be extruded in both sol and gel states through a 26-gauge needle, confirming its suitability for injection. Under an AMF, it exhibited robust heating performance, reaching the thermal ablation range within 3 min. The temperature could be tuned by varying the SPION concentration and dose, and the heating performance remained stable after 5 weeks of storage at 4 °C. Thermal ablation therapeutic outcomes were evaluated in both *ex vivo* and *in vivo* colorectal cancer xenograft mouse models. In *ex vivo* studies, a 15 min treatment reduced tumor volume by 30% immediately post-treatment, while *in vivo*, a single 20 min treatment resulted in a 69% reduction in tumor volume after 2 days. Overall, the magnetic hydrogel provides an alternative for minimally invasive or noninvasive treatment of localized rectal cancer, either for preoperative tumor downsizing or in cases where standard therapies such as surgery or chemoradiotherapy are not feasible.

## Materials and Methods

### SPION Suspension Preparation

The flame synthesis and physicochemical characterization of 23 wt % silica-coated Mn_0.6_Zn_0.4_Fe_2_O_4_ nanoparticles have been described in detail elsewhere.^8^ SPIONs were dispersed in Milli-Q water at 55 mg/mL using a Vibra-Cell cuphorn sonicator (Sonics, Newton, CT, USA) operating at 90% amplitude for 5 min, with 5-s vortexing intervals every minute. Zeta potential of the aqueous SPION suspension was measured by electrophoretic light scattering (Litesizer 500, Anton Paar, Austria) at 25 °C. The Smoluchowski approximation was used to calculate the zeta potential.

### SPION Cytotoxicity

The cytotoxicity of SPIONs was evaluated in CRC cell lines. SW480 (ATCC CCL-228), HT29 (ATCC HTB-38) and Caco-2 (originally obtained from American Type Culture Collection) were cultured in complete cell culture media composed of Dulbecco’s modified Eagle’s medium supplemented with 1% (v/v) l-glutamine (ThermoFisher Scientific), 10% (v/v) fetal bovine serum (ThermoFisher Scientific), 1% (v/v) nonessential amino acids (ThermoFisher Scientific) and 1% (v/v) penicillin/streptomycin (ThermoFisher Scientific). Cells were maintained at 37 °C in a humidified incubator supplied with 10% CO_2_ and were used for experiments within 10 passages after thawing. Cells were seeded into 96-well plates at a density of 155,000 cells/cm^2^ in 200 μL of culture medium. After allowing cells to adhere for 24 h, they were exposed to nanoparticles at different concentrations (0.1, 0.2, 0.4, 0.6, 0.8, and 1 mg/mL) for 24 h. Cell culture medium alone and 0.22% (v/v) sodium dodecyl sulfate were used as negative and positive control, respectively. Cell viability was determined using the CellTiter-Glo luminescent cell viability assay (Promega, USA) according to the manufacturer’s protocol.

### Developmental Toxicity of SPIONs in Zebrafish Embryos

The experiments were conducted with ethical approval from the Swedish Board of Agriculture (permit number: Dnr 5.8.18–10526/2024). AB wild-type zebrafish were maintained at the Centre for In Vivo (CIV), Uppsala University, Sweden. Adults and embryos were housed according to standard procedures.^41^ Embryos were collected and maintained in E3 medium containing 5 mM NaCl, 0.17 mM KCl, 0.33 mM CaCl_2_, 0.33 mM MgSO_4_, and 2 mM HEPES.

A SPION suspension stock was prepared by sonication of a 20 mg/mL aqueous stock using a cuphorn sonicator operating at 90% amplitude for 5 min, with 5-s vortexing between each minute. The stock suspension was then diluted in PBS to obtain final concentrations of 0.1, 0.5, 1, 5, and 10 mg/mL. At 2 days postfertilization (dpf), embryos were anesthetized in tricaine working solution (4 g/L stock, adjusted to pH 7.2 with 1 M Na_2_HPO_4_), microinjected using Narishige IM-31 injector and Borosilicate Glass Capillaries (World Precision Instruments, Lot Number 2604324) with 2 nL of each SPION suspension into the duct of cuvier. PBS was used as a negative control. Following injection, embryos were imaged at 1- and 3-days postinjection using a VAST BioImager (Union Biometrica, USA) under anesthesia. The body, eye, pericard, and swim bladder areas were quantified using a deep-learning method.^42^

### Magnetic Hydrogel Preparation

Pluronic F127 hydrogel (PF127, Sigma-Aldrich, Sweden) was prepared using the cold method.^43^ A 35% (w/v) PF127 stock solution was obtained by dissolving PF127 powder in cold Milli-Q water under continuous magnetic stirring at 4 °C until a clear solution was formed. The 22% PF127 hydrogel used throughout the study was prepared by diluting the stock solution with Milli-Q water. Magnetic hydrogel was prepared by mixing the 35% PF127 stock solution with the 55 mg/mL SPION suspension at 4 °C via vortexing. The final PF127 concentration in the magnetic hydrogel was maintained at 22%, while the SPION concentration varied between 5 and 20 mg/mL, depending on the experiment. Throughout the main text, the term “magnetic hydrogel” refers to hydrogel containing 20 mg/mL SPIONs, and “PF127” refers to 22% PF127 hydrogel unless otherwise specified. The pH of the PF127 and the magnetic hydrogel was 6.8 and 6.6, respectively. Zeta potential of the PF127 hydrogel was measured by electrophoretic light scattering (Litesizer 500, Anton Paar, Austria) at 20 °C to ensure that the sample remained in the sol state. The Smoluchowski approximation was used to calculate the zeta potential.

### Characterization of Magnetic Hydrogel

Magnetic and PF127 hydrogels (both in gel state) were plunge-frozen in liquid ethane using a Vitrobot (Mark IV, Thermo Fisher Scientific) prior to SEM imaging. To freeze the samples, 4 μL of liquid sample was applied to an EMR Holey Carbon TEM grid (Au support, 200 mesh), which was previously glow discharged at 25 mA for 2 min. The grid was then blotted from the backside to remove excess material and then mounted at 37 °C for 1 min to trigger the sol–gel transition before being plunged into liquid ethane. Frozen samples were transferred to an Aquilos 2 cryo-FIB/SEM (Thermo Fisher Scientific) and sublimated overnight at −100 °C under 4 × 10^–7^ mbar to reveal the hydrogel’s pore structure. Following sublimation, samples were cooled to −190 °C and sputter-coated with platinum to prevent charging (30 mA, 15 s). SEM imaging was performed with an Everhart-Thornley secondary electron detector at 3 kV and 13 pA, with a dwell time of 50 ns and 64-frame averaging.

Feret’s diameter was measured manually in FIJI software following an established protocol.^44^ Briefly, the SEM images were first calibrated to the scale bar in the original images and the threshold was adjusted to highlight only the pores. Particle analysis was then conducted using the “Analyze Particles” function, with a size range from 0.001 to infinity and circularity from 0 to 1. Feret’s diameter values were generated automatically via the “Display Results” option.

Thermal properties of magnetic and PF127 hydrogels were analyzed using differential scanning calorimetry (DSC, Q2000, TA Instruments, Delaware, USA). The samples were placed in aluminum pans (TA Instruments, USA) and hermetically sealed with aluminum lids (TA Instruments, USA). A sealed empty pan was used as reference. The DSC scans were performed from 40 to 100 °C at a heating rate of 1 °C/min under a nitrogen atmosphere.

Thermogravimetric analysis (TGA) was performed using a TGA 550 instrument (TA Instruments, Delaware, USA) to determine the thermal decomposition profiles of the hydrogel formulations. Samples were placed in a platinum pan and heated from 20 to 900 °C at a rate of 2 °C/min under a nitrogen flow of 20 mL/min. Rheological properties of the magnetic and PF127 hydrogels were measured using a rotational rheometer (ARES G-2 Rheometer, TA Instruments, Delaware, USA) equipped with a 25 mm parallel plate geometry. The gap between plates was set to 0.7 mm. All samples were stored at 4 °C to maintain their sol state prior to testing, and 100 μL was loaded onto the temperature-controlled bottom plate (Peltier plate system). A solvent trap was used throughout all measurements to prevent sample dehydration. Amplitude oscillation sweeps at oscillation strain ranging from 0.01% to 100% were first performed at 37 °C to determine the LVR. Based on this, an oscillation strain of 0.1% was selected for the temperature sweep measurements. Temperature sweeps were carried out from 4 to 80 °C with a temperature increment of 2 °C per step, at constant strain of 0.1%, and an angular frequency of 10 rad·s^–1^. Oscillation amplitude measurements were then performed with strain varying from 0.01% to 100% at 37 °C with a constant frequency of 1 Hz.

The stability of the magnetic hydrogel was assessed by monitoring its rheological (temperature sweep) and heating profiles after storage at 4 °C for one month. Measurements were performed at weeks 1 and 5 using the same protocols as described previously.

### Heating Performance Measurement

The heating performance of the magnetic hydrogel was measured using the magneTherm system (nanoTherics Ltd., United Kingdom) equipped with a circulating water jacket to maintain the sample surrounding temperature at 37 °C. SPIONs at 5, 10, 15, and 20 mg/mL were loaded into a 15 mL Falcon tube and placed in the center of the water jacket, surrounded by a 9-turn coil (diameter = 44 mm). After temperature stabilization at 37 °C, an AMF (*f* = 590.7 kHz, *H* = 14 mT) was applied for 30 min. The strength of the applied AMF field was within the clinical safety limit known as the Brezovich criterion.^45^ Temperature was continuously monitored using a fiber optic probe (OSENSA, Canada). The temperature difference (Δ*T*) between the start and the end of the measurement was calculated according to eq 1.

1 $$\Delta T=T_{30\min}-T_{0\min}$$

Volume-dependent heating performance was also assessed using 100–500 μL of magnetic hydrogel, following the same protocol.

### Texture Profile Analysis and Tissue Bioadhesion

The texture profile of the hydrogel samples was measured using a TA-XT plusC texture analyzer (Stable Micro Systems Ltd., Godalming, U.K.) equipped with a 45° conical probe *perspex* (p/45c). A plastic cylinder-shaped vial with a diameter of 17.4 mm containing 2 cm of hydrogel samples (equivalent to 4 mL of gel) was fixed on the stage with a double-sided tape. Measurements were initiated with a trigger force of 0.03 N. Upon detecting the trigger force, the probe compressed the gel at 1 mm/sec for 7 mm before being withdrawn at the same speed. A commercial rectal hemorrhoid gel (AC3, Meda AS, Sweden) was tested for comparison. Hardness was defined as the maximum compression force (positive peak), and adhesiveness as the negative area under the curve during the withdrawal phase.

Bioadhesion was assessed using porcine rectal tissue obtained from a local slaughterhouse and analyzed with the same texture analyzer. The experimental setup followed a published protocol.^46^ Cylindrical steel adhesion probes (diameter = 13 mm) were used. Porcine rectal tissues were punched out using a 12 mm diameter circular punching tool and immobilized on the upper probe using a double-sided tape. Magnetic hydrogel (200 μL) was applied on the lower probe and heated above its gelation temperature. The lower and upper probes, tissue and hydrogels were kept in an oven at 37 °C until testing. During measurement, the upper probe (with tissue) contacted the gel with a 0.01 N trigger force, then compressed it at 1 mm/s to a force of 0.05 N, which was held for 10 s. The probe was withdrawn at 0.1 mm/s, and the work of adhesion was calculated as the negative area under the force–time curve during withdrawal. Sample temperature was monitored with a thermal camera to ensure it remained above the gelation point. The same procedure was repeated for PF127 hydrogel and AC3 gel controls.

### MC38 Xenograft Model

Four-month-old male and female C57Bl/6 mice were used for both *ex vivo* and *in vivo* experiments. Animals were housed in the animal house facility of the Biomedical Sciences Research Center Alexander Fleming under specific pathogen-free conditions with controlled temperature (22 ± 2 °C), humidity (55 ± 10%), and a 12 h light/dark cycle. All animal experiments were approved by the Institutional Animal Care and Use Committee of BSRC Fleming (protocol number: 1175208) and conducted in accordance with European and national guidelines for the care and use of laboratory animals.

Wild-type mice were injected subcutaneously in both flanks with 500,000 MC38 murine colon adenocarcinoma cells per site. Tumor growth was monitored every 2 days using a digital caliper. Tumors were considered ready for experimentation when they reached approximately 7–9 mm in size, typically 7–9 days postinjection.

### *Ex vivo* Thermal Ablation

Mice were euthanized by CO_2_ inhalation, and tumors were immediately excised and placed in cold PBS. Tumors were transferred to Millicell cell culture inserts (0.4 μm pore size, 30 mm diameter, Merck Millipore Ltd., Germany), positioned in 35 mm Petri dishes containing 1 mL of cell culture medium (high-glucose DMEM supplemented with 10% fetal bovine serum, 100 U/mL penicillin, 100 μg/mL streptomycin, 2 mM l-glutamine, 50 μg/mL gentamicin, 1 μg/mL amphotericin B, and 1% nonessential amino acids) to maintain a hydrated air–liquid interface. For treatment, 3 mL of magnetic or PF127 hydrogel was applied to fully cover each tumor. Tumors were positioned at the center of a 9-turn coil to receive maximal AMF exposure (*H* = 14 mT, *f* = 590.6 kHz) for 15 min. Temperature was monitored using an infrared thermal camera (Fluke Ti480 Pro, Fluke Europe, The Netherlands). Tumor length (*L*) and width (*W*) were measured before and after AMF treatment with a digital caliper, and tumor volume (*V*) was calculated according to eq 2.

2 $$V=\frac{L\times W\times W}{2}$$

Following AMF exposure, tumors were washed with PBS, fixed in 10% formalin for at least 5 h at 4 °C, and transferred to PBS containing 0.05% sodium azide for storage at 4 °C until histological analysis.

### *In Vivo* Thermal Ablation

On day 0, mice were weighed and anesthetized with a solution of ketamine (200 mg/kg), xylazine (15 mg/kg), and atropine (0.05 mg/kg) at a dose of 5 μL/g of body weight. Prior to administration, both the magnetic hydrogel and PF127 were sterilized under UV light overnight. Following anesthesia, 200 μL of magnetic hydrogel or PF127 hydrogel was injected intratumorally into one of the dorsal tumors. Tumor height and width were measured post-hydrogel injection using a digital caliper. Mice (*n* = 16; females = 7, males = 9) were positioned in a 3D-printed 37 °C water jacket and centered within a 9-turn coil to ensure maximal AMF exposure (*H* = 14 mT, *f* = 590.6 kHz) for 20 min. Tumor temperature was monitored in real time using an infrared thermal camera. Following AMF exposure, mice were placed on a heating pad and administered an anesthesia antidote to aid recovery. A separate control group (*n* = 6; female = 1, males = 5), injected with either magnetic hydrogel or PF127 hydrogel into one of the tumors, underwent the same experimental procedure, excluding AMF exposure.

Two days post-treatment, tumor length and width were measured again prior to sacrifice. Tumor volume (*V*) was calculated according to eq 2. Then, the tumors were then excised, washed in PBS, and fixed in 10% formalin for at least 5 h. After fixation, tumors were washed again in PBS and stored in PBS containing 0.05% sodium azide for histological analysis.

The kidneys, spleen, and liver were also dissected from nontreated control mice (*n* = 3) and mice that underwent thermal ablation (*n* = 4). The organs were washed with PBS, gently blotted dry, and weighed before being snap-frozen in liquid nitrogen. The tissues were homogenized using a T10 Basic homogenizer (IKA-Werke GmbH & Co. KG, Germany) in 5 mL of 1 M NaOH and incubated at 60 °C overnight. Subsequently, 0.5 mL of 12 M HCl was added to each sample. Prior to iron concentration measurement, the samples were filtered through a 0.45 μm filter and preserved with 65% DuoPur HNO_3_. Iron concentrations were determined by inductively coupled plasma mass spectrometry (Agilent 7900, USA) by Eurofins (Sweden).

### Tumor Histology

Fixed tumors stored in PBS containing 0.05% sodium azide were washed in PBS, processed using the Spin Tissue Processor (Leica TP1020) and embedded in paraffin. Tissue sections were obtained using a microtome (SLEE medical) at 4 μm and stained with hematoxylin and eosin using the Leica ST5010 XL autostainer. H&E-stained tissue sections were imaged using an Olympus Slide Scanner VS200 (20X lens) and the OlyVIA (Ver.2.9.1) software.

## Supplementary Material



## Notes

Zeta potential of PF127 hydrogel and SPION suspension (Table S1); body, eye, and pericardial area quantification in zebrafish embryos at 1 dpi (Figure S1); temperature-dependent storage and loss moduli of PF127 and magnetic hydrogels (Figure S2); oscillatory amplitude sweep of PF127 hydrogel at 37 °C (Figure S3); iron distribution in kidneys, spleens, and liver of control and magnetic hydrogel–treated mice (Figure S4) (PDF)
