# Supplementary material for: Thermoresponsive Magnetic Hydrogel for Local Thermal Ablation Treatment of Rectal Cancer
Source: ACS Appl Mater Interfaces. 2026 Jan 2;18(1):914–26. doi: 10.1021/acsami.5c22174 (PMC12781055; doi:10.1021/acsami.5c22174)
Supplement: Supplementary file 1 [file am5c22174_si_001.pdf]

Supporting Information:

Thermoresponsive magnetic hydrogel for local thermal ablation treatment of rectal cancer

Yuming Zhang[1], Christina Paraskeva[2], Isha Shaffir[1], Marco Tjakra[3],
Vasiliki Koliaraki[2], Alexandra Teleki[1,*]

[1]Department of Pharmacy, Science for Life Laboratory, Uppsala University, 75123 Uppsala, Sweden, [2]Institute for Fundamental Biomedical Research, Biomedical Sciences Research Center 'Alexander Fleming', 16672 Vari, Greece, [3]Department of Pharmacy, Uppsala University, 75123 Uppsala, Sweden

*Corresponding author: alexandra.teleki@scilifelab.uu.se

Table S1. Zeta potential of PF127 hydrogel and SPION water suspension. All data are expressed as mean ± SD (n≥3).

	**Zeta potential (mV)**
22% PF127	-0.03±0.23
1 mg/mL SPION water suspension	-40.78±5.76

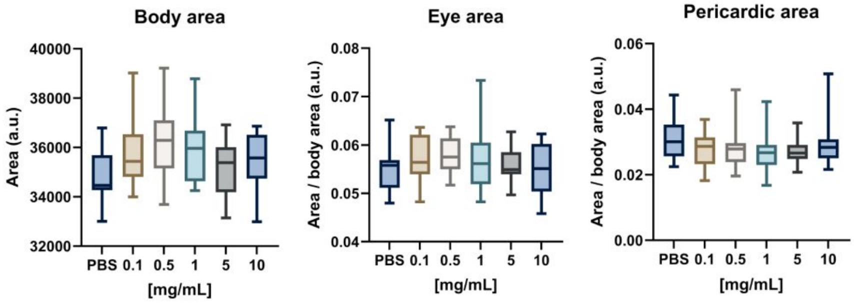

Figure S1. Quantification of body, eye, and pericardial areas at 1 dpi ($n \geq 11$). Data are expressed as mean ± standard deviation. No significant differences were detected (all $p > 0.05$).

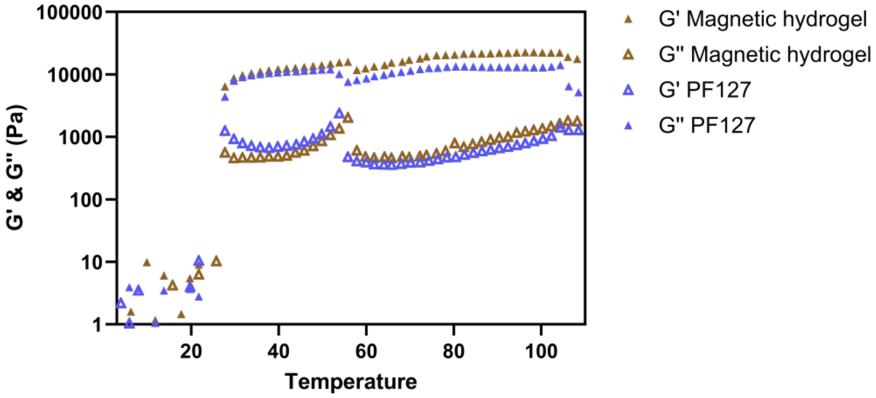

Figure S2. G’ and G’’ as a function of temperature at a constant oscillation strain of 0.1% for the PF127 (blue symbols) and magnetic hydrogels (brown symbols).

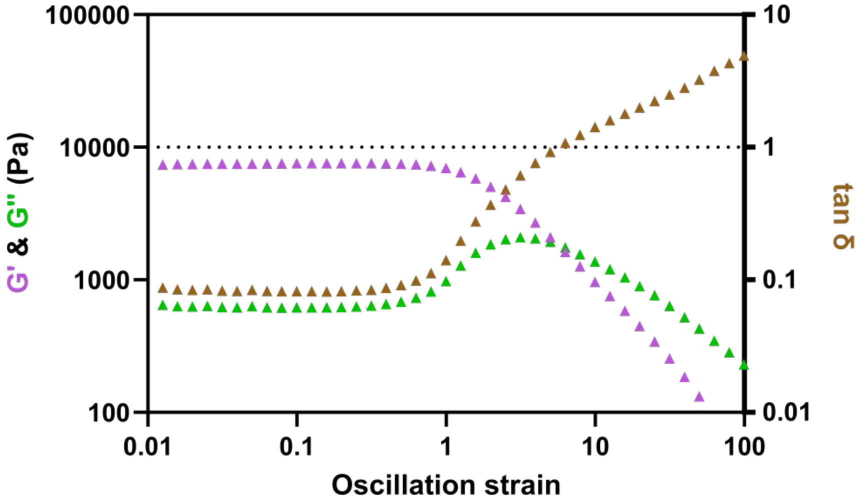

Figure S3. Oscillatory amplitude sweep of PF127 hydrogel at 37°C at a constant frequency of 1 Hz.

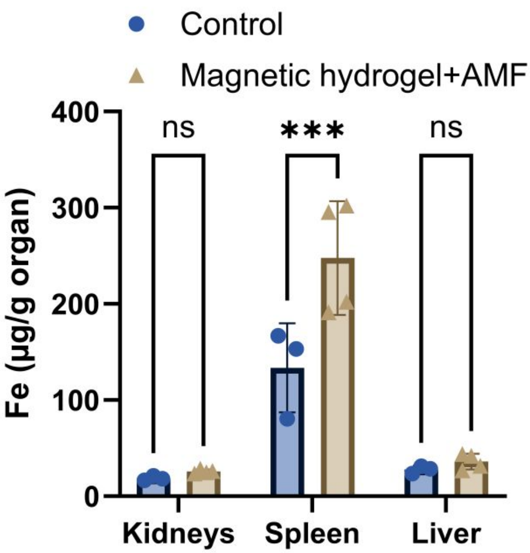

Figure S4. Fe distribution in kidneys, spleens, and liver from non-treated control mice and mice that underwent magnetic hydrogel thermal ablation (n ≥ 3).
